# Simulated Gastric Digestion and In Vivo Intestinal Uptake of Orally Administered CuO Nanoparticles and TiO_2_ E171 in Male and Female Rat Pups

**DOI:** 10.3390/nano11061487

**Published:** 2021-06-04

**Authors:** Ninell P. Mortensen, Maria Moreno Caffaro, Shyam Aravamudhan, Lakshmi Beeravalli, Sharmista Prattipati, Rodney W. Snyder, Scott L. Watson, Purvi R. Patel, Frank X. Weber, Stephanie A. Montgomery, Susan J. Sumner, Timothy R. Fennell

**Affiliations:** 1 RTI International, 3040 E Cornwallis Road, Research Triangle Park, NC 27709, USA; mmoreno@rti.org (M.M.C.); rsnyder@rti.org (R.W.S.); slwatson@rti.org (S.L.W.); ppatel@rti.org (P.R.P.); fxw@rti.org (F.X.W.); fennell@rti.org (T.R.F.); 2 Joint School of Nanoscience and Nanoengineering, 2907 East Gate City Blvd., Greensboro, NC 27401, USA; saravamu@ncat.edu (S.A.); lpbeerav@uncg.edu (L.B.); sprattip@aggies.ncat.edu (S.P.); 3 Department of Pathology and Laboratory Medicine, The University of North Carolina at Chapel Hill, Chapel Hill, NC 27599, USA; stephanie_montgomery@med.unc.edu; 4 UNC Nutrition Research Institute, The University of North Carolina at Chapel Hill, 500 Laureate Way, Kannapolis, NC 28081, USA; susan_sumner@unc.edu

**Keywords:** rat pups, simulated gastric digestion, tissue uptake, ENM stability, CuO nanoparticles, food grade TiO_2_ E171, immune cells, early life exposure, oral administration

## Abstract

Oral exposure to nanoparticles (NPs) during early life is an understudied area. The goals of this study were to evaluate the effect of pre-weaned rat gastric fluids on 50 nm CuO NPs and TiO_2_ E171 in vitro, and to evaluate uptake in vivo. The NP uptake was studied in vivo in male and female Sprague-Dawley rat pups following oral administration of four consecutive daily doses of 10 mg/kg CuO NPs, TiO_2_ E171, or vehicle control (water) between postnatal day (PND) 7–10. Rat pups were sacrificed on either PND10 or PND21. Simulated digestion led to dissolution of CuO NPs at the later ages tested (PND14 and PND21, but not PND7). In vivo intestinal uptake of CuO NPs and TiO_2_ E171 was observed by hyperspectral imaging of intestinal cross sections. Brightfield microscopy showed that the number of immune cells increased in the intestinal tissue following NP administration. Orally administered NPs led to low intestinal uptake of NPs and an increase in immune cells in the small and large intestine, suggesting that oral exposure to NPs during early life may lead to irritation or a low-grade inflammation. The long-term impact of increased immune cells in the intestinal tract during early life is unknown.

## 1. Introduction

Engineered nanomaterials (ENMs), including nanoparticles (NPs), are increasingly being incorporated in consumer products or may enter unintentionally during production or processing. Food grade titanium dioxide (TiO_2_ E171) is a food additive used as a white pigment and brightening agent and has been reported in food products at concentrations between 0.0002–5.4 mg Ti/g product [1,2]. The European Food Safety Authority (EFSA) estimated that the mean daily consumption of TiO_2_ E171 ranges between 0.2–1.9 mg/kg body weight (bw) per day for infants, 0.6–9.2 mg/kg bw/day for toddlers, and 0.9–10.4 mg/kg bw/day for children [3]. CuO NP is used in the agricultural sector [4,5] and is being explored as an antimicrobial agent in food packaging [6,7,8]; it may thus unintentionally end up in food products. Children represent a vulnerable population because perturbations in cell growth and signaling can disrupt temporally sequenced developmental processes leading to long-term functional deficits. Oral exposure to NPs during early life is an understudied area, and more knowledge is needed to understand their impact in developing animals and what health risk NPs pose.

Early life exposure studies to NPs are few and the short- and long-term consequences of exposure are not well understood. Direct exposure studies of postnatal rodents have been reported for subcutaneous (s.c.) injection [9], intraperitoneal (i.p.) injection [10], intranasal instillation [11,12], inhalation [13], and oral [14] exposure. Since the rat gastrointestinal tract undergoes significant anatomical and biochemical changes in the first three postnatal weeks, including gut closure [15,16,17,18,19] and gradually lowering of the gastric pH [20], the developing gastrointestinal tract in rats could be more vulnerable to NPs. The gastric pH steadily decreases from pH 7 in newborn rat pups (bland phase) to pH 6 around postnatal day (PND) 14 (transition phase) before it reaches pH 4 around weaning at PND 21 (acidic phase) [20]. During the bland phase, mucus producing cells are absent from the rat stomach and only nonproductive parietal cells are present [20]. At PND 14, parietal cells (producing HCl) and chief cells (producing pepsin) have started to develop [20]. In addition to the dramatic changes taking place in the gastrointestinal tract, virtually all major organs undergo significant development after birth, including the brain and central nervous system [21,22], the liver [23,24,25], and the immune system [26,27].

An important aspect of oral exposure is understanding how gastrointestinal digestion might change the physiochemical properties (PCPs) of NPs. Simulated human digestion studies demonstrate changes in the PCPs of AgNP [28], SiO_2_ NP [29], Cu NP, [30], ZnO NP [31], and Fe_2_O_3_ NP [32], but not TiO_2_ NP [30]. The biological impact of NPs in vitro can also be altered by simulated digestion [28,30,33,34,35]. TiO_2_ NP is a very stable nanomaterial with a low level of dissolution [30], while CuO NP is a less stable nanomaterial and more readily undergoes dissolution [36]. While simulated rat digestion models have been reported in the literature [37,38,39], no studies, so far, have explored the biotransformation of metal and metal oxide NPs in adult or developing animals.

In the study presented here, male and female rat pups were orally administered CuO NP or food grade TiO_2_ E171 between PND 7–10. At this developmental stage prior to weaning, the gastrointestinal tract has not yet reached the adult anatomical and physiological stages, and “gut closure” has not been completed [40,41]. The oral route was chosen in order to investigate ingestion of NPs and the interaction with the intestinal tract. NPs were orally administered daily as four consecutive doses of 10 mg/kg. The dose selection was based on the estimated daily consumption of TiO_2_ [2] and reported TiO_2_ concentrations in food products [1]. Based on these data, 10 mg/kg for TiO_2_ E171 was chosen for this exploratory investigation. The selected dose might be high for CuO NP; however, since the aim of this study is to provide a comparison between the two NPs the same concentration was used for both.

This study was conducted as part of a National Institute of Environmental Health Sciences (NIEHS) Consortium for Nanotechnology Health Implications Research (NHIR) which provided the CuO NP and TiO_2_ E171. The biotransformation and stability of the two NPs were characterized following simulated in vitro gastric digestion for different developmental ages. During the in vivo study, body weights were recorded, and weight gain was calculated. Following termination, organ-to-bw ratios were calculated, NP uptake was evaluated using enhanced darkfield microscopy (EDM) and hyperspectral imaging (HSI) in Alcian Blue Periodic Acid Schiff (AB-PAS) stained cross sections of the small and large intestine, and the number of immune cells were counted in the intestinal tract by brightfield microscopy of H&E stained Swiss roll tissue sections. The results presented here provide a basic understanding of the biotransformation, uptake, and biological impacts of CuO NP and TiO_2_ E171 in the intestinal tract due to early life exposure to these NPs.

## 2. Materials and Methods

### 2.1. Nanomaterials and Chemicals

The 50 nm CuO NP (Sigma-Aldrich, St. Louis, MO, USA) and TiO_2_ E171 (Pronto foods Co., Chicago, IL, USA) was procured, comprehensively characterized, and provided by the Engineered Nanomaterials Resource and Coordination Core (ERCC) as part of the NIEHS-NHIR Consortium.

Solvents and chemicals for in vitro digestion studies included sodium chloride (NaCl), hydrochloric acid (HCl), sodium bicarbonate (NaHCO_3_), and monobasic potassium phosphate (KH_2_PO_4_), procured from Fisher Scientific (Suwanee, GA, USA). Mucin was purchased from Sigma-Aldrich). Pepsin from porcine gastric mucosa was purchased from Thermo Fisher Scientific (Waltham, MA, USA). Solvents for tissue fixation included 10% buffered formalin (Thermo Scientific™ Richard-Allan Scientific, Pittsburgh, PA, USA), ethanol (Decon Laboratories, Inc, King of Prussia, PA, USA), xylene (Fisher Scientific, Suwanee, GA, USA) and from Fisher Scientific (Oak Ridge, TN, USA) acetic acid, methanol, and chloroform. Nitric acid for Inductively Coupled Plasma Optical Emission Spectrometry (ICP-OES) analysis was purchased from Fisher Scientific (Suwanee, GA, USA). Solvents and chemicals for inductively coupled plasma mass spectrometry (ICP-MS) included Multi-Element (ME) solution, Ti stock, Ho stock, and Pr stock from SCP Science (Baie-D’Urfé, QC, Canada), Trace Metal Grade HNO_3_ (Fisher, Fair Lawn, NJ, USA), and Trace Metal Grade H_2_O_2_ (EMD, Burlington, MA, USA).

### 2.2. In Vitro Gastric Digestion

Simulated gastric digestion of CuO NP and TiO_2_ E171 was conducted for three developmental gastric phases; bland phase (~PND 7), transitional phase (~PND 14), and acidic phase (~PND 21) for 1, 2, and 4 h (Table 1). The acidic phase was performed as published by Chen et al. [37]. The pH of the rat stomach is neutral at birth and through PND 7, then drops to pH 6 at PND 14, and at PND 21 has reached pH 4 [20]. The bland phase is due to the lack of HCl production the first 7 days [20]. Pepsin production starts at PND 14 [20] and is mimicked by using half of the adult pepsin level. There are no mucus producing cells at PND 7 [20], so mucin is only included in the transitional and acidic phase. Incubation was carried out at 37 °C at a concentration of 0.25 mg/mL NP. Multiple time points were tested for each gastrointestinal solution reflecting a range of possible gastric transit times. Digested NPs were characterized by dynamic light scattering (DLS), scanning electron microscope (SEM) and ICP-OES analysis, as described below.

### 2.3. Nanoparticle Stability in Tissue Fixative Solutions

The stability of CuO NP and TiO_2_ E171 in the solutions used for tissue fixative and preparation for tissue sectioning was investigated at room temperature at a concentration of 0.25 mg/mL. The fixative solutions tested were 10% buffered formalin, Bouin’s fixative (50% ethanol/5% acetic acid in dH_2_O), and Metha-Carnoy’s solution (60% methanol, 30% chloroform, 10% glacial acetic acid). Different ethanol solutions (70%, 90%, and 100%) used in the preparation of tissue for sectioning were also tested, as was xylene. Two time points were tested for each solution reflecting the possible range of time each processing step can take. NPs stability was characterized by DLS, SEM, and ICP-OES analysis, as described below.

### 2.4. Nanoparticle Characterization

Pristine NPs were characterized using transmission electron microscope (TEM) (LIBRA^®^120, Carl Zeiss Microscopy, Peabody, MA, USA), and NP in vivo dose suspension characterized using DLS (Malvern Zetasizer Nano-ZS, Malvern Panalytical, Westborough, MA, USA), zeta-potential, and nanoparticle tracker (NTA) (NanoSight LM10, Malvern Panalytical). For TEM imaging samples were prepared by placing a drop of NP suspension at low density on formvar-coated TEM grids (Ted Pella, Redding, CA, USA), followed by rinsing in DDH_2_O and finally, drying at room temperature. Digested NPs were characterized using DLS, SEM, and ICP-OES (see below). SEM was performed using a Zeiss Auriga field emission scanning electron microscope (FESEM) (Carl Zeiss Microscopy, White Plains, NY, USA) at 4 or 5 kV accelerating voltage and a beam current of 10 µA.

### 2.5. Inductively Coupled Plasma Optical Emission Spectrometry (ICP-OES) Analysis of Digested NPs

For each sample, 5 mL NPs in gastric fluid was filtered through a 5000 molecular weight cut off (MWCO) centrifuge filter (Sartorius Vivaspin, Goettingen, Germany) (cut off < 2 nm) at 8000 rpm for 20 min. Undigested NPs in gastric fluids were filtered out in the process. The filtrate was digested in 3% nitric acid overnight and analyzed by ICP-OES. The filtered NPs were also separately digested in 3% nitric acid overnight and analyzed by ICP-OES. All ICP-OES analyses were performed in triplicate on at least three samples of the same type. Following the recommendation of International Union of Pure and Applied Chemistry (IUPAC), detection limit was calculated in terms of three times the standard deviation (98% confidence) of 10 replicates of blank measures. Limit of detection (LOD) was 5 ng Cu mL^−1^ and 50 ng Ti mL^−1^.

### 2.6. Nanoparticle In Vivo Dose Formulation

For in vivo exposure studies, NPs were formulated as dosing suspension of 2 mg/mL in filtered deionized water and sonicated in a cup horn sonicator (Ultrasonic Liquid Processor S-4000, Misonix Inc., Farmingdale, NY, USA) by adapting published protocols [42,43]. To determine the critical delivered energy (DSEcr [J/mL]), DLS was used to measure the hydrodynamic diameter every 2–5 min, until the change in NP diameter was less than 5%. The DSEcr for the dosing solution was determined to be 2886 J/mL for CuO NP and to be 1690 J/mL for TiO_2_ E171. Dosing solutions were prepared freshly every day in deionized water. The stability of the dispersed NPs was measured in water after 0 h and 4 h by DLS, zeta-potential, and NTA. The criterium for stability was that the hydrodynamic diameter should only change <30% in a time course of 4 h.

### 2.7. Housing and Dose Administration

Lactating Sprague Dawley rats with their standardized litter of five male and five female pups age PND 2–3, were obtained from Charles River Laboratories (Raleigh, NC, USA). All rats were acclimated 4–5 days prior to start of dosing. Three litters, a total of 15 female and 15 male pups, were included in each dosing group. Animal care and handling were done in compliance with the Guide for the Care and Use of Laboratory Animals [44] and approved by the Institutional Animal Care and Use Committee (IACUC) of Mispro Biotech, Research Triangle Park, NC, USA. Lactating rats and their litters were housed in individual polycarbonate cages and fed LabDiet 5058 Breeder Diet (LabDiet, Durham, NC, USA) and Durham City water from a reverse osmosis system was provided ad libitum. The temperature was maintained at 72 ± 3 °F, the relative humidity at 30–70% in the animal room with a 12:12 light cycle.

A daily dose of 10 mg/kg NPs CuO NP or TiO_2_ E171 was orally administered to the pups between PND 7–10. An equal volume of deionized water was administered as the vehicle control. Prior to every dose administration each pup was weighed, and the appropriate volume of the dosing solution (based on body weight) drawn into the syringe. A stainless steel 22G ball-tipped gavage dosing needle and an appropriately sized syringe was used for gavage dosing. One male and one female from each of the three litters were sacrificed four hours after the administration of the fourth and last dose on PND 10. The remaining four male and four female per litter were sacrificed on PND 21. The pups were euthanized by live decapitation. Dams were sacrificed by overexposure to CO_2_.

The liver and brain were collected and weighed at PND 10 and PND 21. Duodenum, jejunum, ileum, and colon from two pups per litter (one male and one female pup) were collected for histopathology at PND 10, four hours after receiving the fourth and last dose.

### 2.8. Histology

For the four intestinal segments (duodenum, jejunum, ileum, and colon), cross sections were collected on the distal part of the tissue and the remaining tissue was prepared by Swiss roll. For cross sections, the proximal 2–3 × 5 mm sections of duodenum, jejunum, ileum, and colon were quickly collected and fixed for histopathology as published by Johansson et al. [45] and slightly modified by Desai et al. [46]. In short, cross sections were fixed in freshly made Metha-Carnoy’s fixative (60% methanol, 30% chloroform, 10% glacial acetic acid) for 3 h at room temperature, followed by transfer to fresh Metha-Carnoy’s fixative overnight at 4 °C. The next day tissues were washed twice in 100% methanol for 30 min and washed twice in 100% ethanol for 20 min. Samples were stored in 100% ethanol at 4 °C until further processing. The remaining duodenum, jejunum, ileum, and colon tissues were prepared using the improved Swiss roll technique, following the published protocol by Bialkowska et al. [47]. In short, each of the four intestinal sections were immediately rinsed with 10 mL of modified Bouin’s fixative (50% ethanol/5% acetic acid in dH_2_O). The rinsed tissues were cut open longitudinally along the mesenteric line and rolled from the proximal to the distal end with the lumen facing up. Tissues were then fixed in 10% buffered formalin for 72 h, washed briefly in deionized water and transferred to 70% ethanol until processed.

Tissues were paraffin-embedded, sectioned at 4 microns thick, and histochemical staining was performed at the Lineberger Comprehensive Cancer Center Animal Histopathology Core, University of North Carolina Chapel Hill (Chapel Hill, NC, USA). Cross sections were stained with Alcian Blue Periodic Acid Schiff (AB-PAS) and Swiss rolls with hematoxylin and eosin (H&E). Histomorphology of the duodenum, jejunum, ileum, and colon were examined by a board-certified veterinary pathologist using an Olympus BX43 light microscopy (Olympus, Waltham, MA, USA).

### 2.9. Evaluation of NPs Present in Intestinal Tissues

Cross sections from NP dosed and control animals were imaged using an enhanced darkfield microscope (EDM) with a hyperspectral imaging (HSI) unit (CytoViva Inc., Auburn, AL, USA). The microscope includes a standard BX41 Olympus microscope, dark field condenser, and hyperspectral imaging unit. Images were first collected using EDM and the software Q-Capture 7 (CytoViva Inc.), followed by HSI using the software Environment for Visualization (ENVI), version 4.8 (Harris Corporation, McLean, VA, USA). Images were collected as previously described using a 60× oil objective [14]. The visual appearance (e.g., color scale) of HSI images does not reflect the spectral data and should be interpreted with care. HSI image features can be enhanced using different computational software filters to aid the visualization by eye. Differences in hue between images has previously been described [48]. A filter takes a valuation of all the red-green-blue channels of the image and renders the end result, so if one image is predominantly tissue with little black background and another image is predominantly black background and less tissue, this can cause a difference. Differences in the lighting or focus of the illuminator between images can also impact the hue, but not the data. As a result, the visual appearance of HSI images might be different for the same tissue from the same treatment group even when the same enhancement filter was selected.

Spectral libraries were built for cross sections of each of the four intestinal segments using at least ten images (five males and five females) and each library was filtered against at least ten images (five males and five females) of the corresponding vehicle control tissue to remove any spectra not unique to CuO NP or TiO_2_ E171 (Supplementary Figure S1). The spectral library for TiO_2_ E171—ileum had to be divided into two spectral libraries (lumen and tissue) since it was otherwise too large for the software to do spectral mapping. The ileum-tissue spectral library was filtered against the ileum-lumen spectral library to ensure spectra unique to TiO_2_ E171 was not double counted. For each dosing group at least five images were collected for each tissue section from three male and three female pups. The filtered libraries were then used to map the location of spectra unique to NPs in each image using the ENVI 4.8 software. The overlay image of mapped spectra on the corresponding hyperspectral image was saved as a tiff file. ImageJ (National Institutes of Health, Bethesda, MD, USA) was used to count the areas of identified spectra in each image for each of the three following tissue areas: Lumen, mucus, and epithelium. The area of NP signals was counted using ImageJ, these could correspond to individual or small aggregates of NPs as previously described [14]. It should be noted, that HSI mapping of unique spectra could, in addition to NP, also capture changes in tissues due to inflammation, apoptosis, necrosis, etc. To ensure that the unique spectra were not caused by inflammation, apoptosis, nor necrosis, pathological evaluation were done under light microscopy and were found to be within normal limits. We therefore assume that the HSI libraries mapped the location of the administered NP.

### 2.10. Evaluation of Immune Cells Present in Intestinal Tissues

The number of granulocytes and intraepithelial lymphocytes (IEL) were counted in bright field microscopy images of H&E stained Swiss roll tissue sections of duodenum and colon. One image per turn in the Swiss roll was collected in the direction from proximal to distal of 2–4 Swiss rolls per gender for each dosing group using 40× air objectives on an Olympus X71 (Olympus, Waltham, MA, USA). Each Swiss roll for the small intestine had between 6–7 turns, while the shorter colon segments had between 4–5 turns, the same number of images were evaluated for each tissue section starting at the proximal end of the Swiss roll. Granulocytes were identified based on morphology as round cells with a bright pink, granulated cytoplasm, and a multi-lobed nucleus. IELs were identified as small, round cells with a condensed chromatin yielding dark nucleus staining and scant cytoplasm located between two epithelium cells.

### 2.11. Inductively Coupled Plasma Mass Spectrometry (ICP-MS) Analysis of Tissues

Quantitative analysis of Cu and Ti in tissues was conducted by ICP-MS, using a modification of EPA 3050B tailored to tissue matrix and limited sample mass. The wet weight of livers from three male and three female rat pups per dosing group sacrificed on PND 10 was collected prior to tissue digestion. The whole organ was processed. Pre-weighed lyophilized tissue was digested in HNO_3_ followed by H_2_O_2_. Internal standards were added to the digested samples. For each set of digestions, method blanks were prepared and analyzed to monitor background Cu and Ti content. The metal concentrations for all samples were analyzed using a Thermo Q ICP-MS (Thermo Q). Quality control samples were processed with the study samples to monitor method performance, including pre-digestion spiked method blanks. The quantification limit was 0.05 ng Cu mL^−1^ digested solution with a linear range from 0.05 to 20.0 ng mL^−1^ and 0.05 ng Ti mL^−1^ digested solution with a linear range from 0.05 to 20.0 ng mL^−1^. Quantitation limits were 2.58 ng Cu g^−1^ tissue and 2.58 ng Ti g^−1^ tissue.

### 2.12. Statistical Analysis

A repeated-measure one-way ANOVA test was used for statistical analysis of body weight (bw) and organ-to-bw ratio between vehicle control and dose groups. To test for significant differences due to NP administration for hepatic metal concentrations and the intestinal number of immune cells and to understand the role of gender, repeated-measure two-way ANOVA tests were conducted with Bonferroni post-hoc tests for multiple comparisons. The statistical analyses were done using the software GraphPad Prism 7.04 (GraphPad Software, San Diego, CA, USA).

## 3. Results

### 3.1. In Vitro Gastric Digestion of Nanoparticles

To investigate the stability of CuO NP and TiO_2_ E171 during simulated gastric digestion at different postnatal ages and gastric transit times, NPs were incubated in in vitro rat gastric fluids for 1, 2, or 4 h, and characterized by DLS, ICP-OES, and SEM. TEM images of pristine CuO NP and TiO_2_ E171 are shown in Supplementary Figure S2. DLS of digested CuO NP and TiO_2_ E171 showed increased hydrodynamic diameter and polydispersity index (Pdi) demonstrating aggregation for both NPs (Table 2), which was confirmed by the SEM images (Supplementary Figure S3). The ICP-OES results showed that CuO NP underwent an increasing degree of dissolution with increasing postnatal age, and corresponding increase in enzyme concentrations and decrease in pH. After 4 h of digestion in bland (~PND 7) and transitional (~PND 14) phases, dissolution was limited to 3.5% and 17% of CuO NP in ionic form, respectively, while dissolution in the acidic phase (~PND 21) occurred at a significantly higher level, resulting in 78% of the CuO NP in ionic form. No detectable dissolution was observed for TiO_2_ E171 under any of the tested conditions.

### 3.2. Nanoparticle Stability in Tissue Fixative Solutions

To ensure that the two NPs did not undergo dissolution during tissue fixation and preparation for tissue sectioning, their stability was tested in the different solutions and at different time points by ICP-OES (Supplementary Table S1). No significant dissolution was observed for either of the NPs, demonstrating that NPs are not lost during sample preparation, but preserved in the tissue sections.

### 3.3. Characterization of In Vivo Dose Solution

Dose solutions of CuO NP and TiO_2_ E171 were formulated at 2 mg/mL in deionized water and characterized by DLS, zeta-potential, and NTA at 0 h and 4 h after formulation to ensure stability from the time of formulation until completion of dosing (Supplementary Table S2). While the zeta-potential for CuO NP was negative in deionized water (4 h: −19.5 ± 0.497) it was close to zero for TiO_2_ E171 (4 h: 0.992 ± 0.518). CuO NP had a high hydrodynamic diameter (1498 ± 102 nm) and PdI (0.521) suggesting aggregation, but the diameter measured by NTA (261 ± 73.0 nm) was considerably smaller. TiO_2_ E171 showed considerably less aggregation (hydrodynamic diameter = 300 ± 9.91 nm; PdI = 0.334; NTA = 250 ± 109 nm). Increasing sonication time did not lower the hydrodynamic diameter for CuO NP. DLS for both dosing solutions were stable for a period of four hours.

### 3.4. Body Weight (bw) and Organ-to-bw Ratio

**Figure 1:**
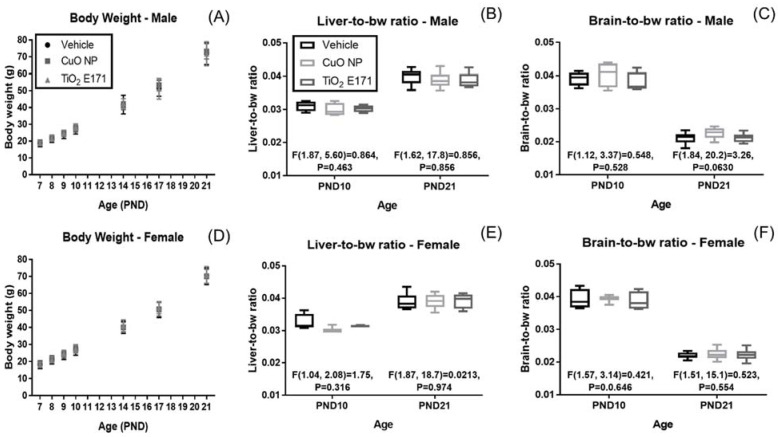
Body weight (bw), liver-to-bw ratio, and brain-to-bw ratio for male (**A**–**C**) and female (**D**–**F**) rat pups dosed between PND 7–10. No changes were observed in body weight as a result of NP oral dosing. While NP administration did not cause in organ-to-bw ratio changes, a clear difference was observed between ages PND 10 and PND 21. Body weight is presented as mean ± standard deviation. Box and whisker plots of organ-to-bw ratio data with statistical F values are shown.

There were no statistical differences detected in body weight (bw), liver-to-bw ratio, or brain-to-bw ratio as a result of NP oral dosing between PND 7–10 as determined by one-way ANOVA (Figure 1).

It is worth noticing that a clear difference in the organ-to-bw ratios was observed between ages PND 10 and PND 21, underlining that the growth in brain and liver happens at different developmental stages. Liver-to-bw ratio were similar for both male and female pups at PND 10 (ranging between 0.030–0.031 and 0.031–0.032, respectively) and increased for both male and female at PND 21 (ranging between 0.039–0.040 and 0.039, respectively). Brain-to-bw ratio was also similar between male and female pups at PND 10 (ranging between 0.038–0.040 and 0.039, respectively) and had decreased at PND 21 (0.021–0.023 and 0.022, respectively).

### 3.5. Intestinal Uptake of Nanoparticles

**Figure 2:**
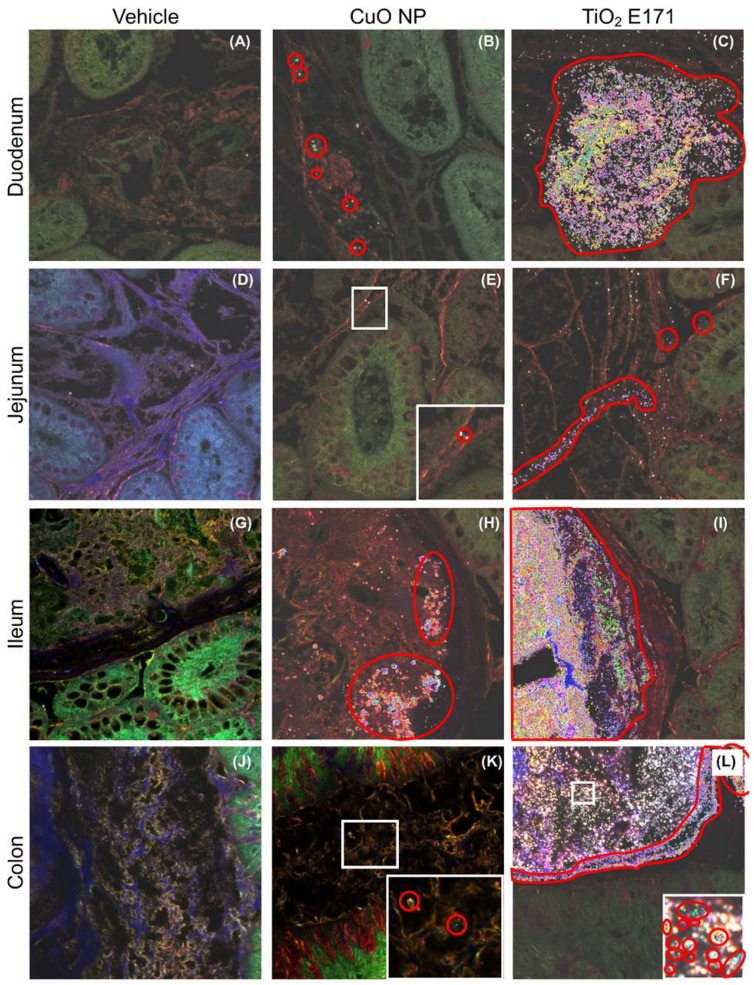
Enhanced darkfield microscopy (EDM) and hyperspectral imaging (HSI) of intestinal cross sections of AB-PAS stained duodenum (**A**–**C**), jejunum (**D**–**F**), ileum (**G**–**I**), and colon (**J**–**L**) tissue for rat pups dosed between PND 7–10 with vehicle control (**A**,**D**,**G**,**J**), CuO NP (**B**,**E**,**H**,**K**), or TiO_2_ E171 (**C**,**F**,**I**,**L**). Mapped NPs (individual pixels) are shown with different colors, and areas with mapped NPs are highlighted with red circles and polygons.

Cross sections of the three small intestinal segments (duodenum, jejunum, and ileum) and the large intestine (colon) were collected four hours post-administration of the last dose (PND 10), fixed with Metha-Carnoy’s fixative preserving the mucus layer, and stained with AB-PAS. The cross sections were used to visualize the intestinal lumen with content, mucus layer, and epithelium for the four intestinal segments. Utilizing a combination of EDM/HSI, the location of spectral profiles unique to each dosing group were mapped for each tissue section and assumed to be NPs. Representative images of the intestinal tissue cross sections are shown in Figure 2. The AB-PAS stained intestinal lumen content appear as loosely packed brown material in the EDM/HSI images, whereas the epithelium and underlaying tissue have a greenish hue. The mucus layer is especially well defined in the colon as a dark brown layer covering the epithelium. NPs were observed in both the lumen, the mucus layer, and at low levels in the underlying epithelium. However, the majority of NPs were observed in the lumen for both CuO NP and TiO_2_ E171, but the amount of TiO_2_ E171 present in the lumen was extensive compared to CuO NP.

**Figure 3:**
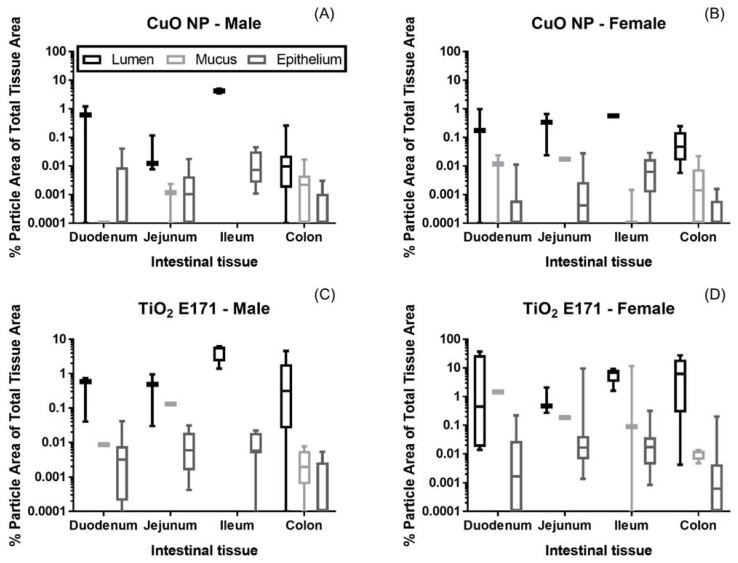
The percent (%) particle area of the total tissue area for CuO NPs (**A**,**B**) and TiO_2_ E171 (**C**,**D**) found in Hyperspectral Imaging (HSI) images of intestinal cross sections of duodenum, jejunum, ileum, and colon for male (**A** + **C**) and female (**B** + **D**) rat pups dosed between PND 7–10 and sacrificed four hours after receiving the last dose. Box and whisker plots of the percent particle area of the total tissue areas are shown.

The area of NPs was calculated for each of the three different tissue areas (lumen, mucus, and epithelium), and expressed as percent particle area of total tissue area (Figure 3). For CuO NP (Figure 3A,B) the highest percentage of particle area was observed in the lumen for both small and large intestines collected from both male and female pups. Mucus areas in the small intestinal were few, but in the large intestine, the percentage of particles was higher in the mucus than in the underlying epithelium tissue. CuO NP were observed at low levels in the epithelium for both the small and large intestine; the percentage of NPs in the epithelium of the small intestinal tract was higher than the epithelium in the large intestinal tract. Also, for TiO_2_ E171 (Figure 3C,D) the highest percentage of particles were observed in the lumen for both small and large intestines collected from male and female pups.

Overall, the percentage of TiO_2_ E171 was higher in all areas of intestinal tissues in female pups than in male pups. As with CuO NP dosed rats, the areas of mucus in the small intestine were few for TiO_2_ E171-dosed rats, but the percentage of particles in the mucus and epithelium of the large intestine were similar. Overall, the percentage of TiO_2_ E171 particles in the epithelium was similar for male and female pups, with a lower level of particles in the epithelium of the large intestine compared to the small intestinal segments.

**Figure 4:**
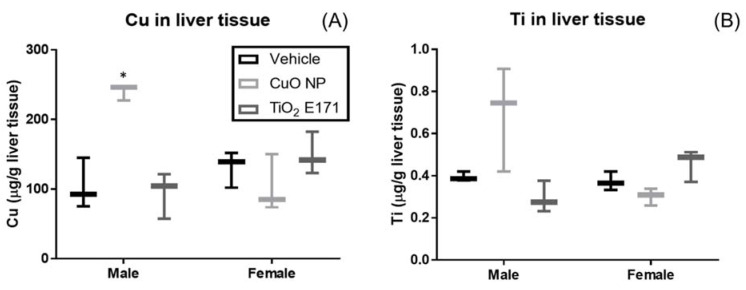
ICP-MS data of liver tissue collected 4 h after administration of the fourth and last dose on PND 10 showing the tissue concentration of Cu (**A**) and Ti (**B**). The concentration of Cu and Ti was determined for all three dosing groups. ICP-MS data presented as mean ± standard deviation. Box and whisker plots of metal concentration are shown. Statistical difference between vehicle control and dosing groups are noted by asterisk(s); *p*-value < 0.05 is noted with one asterisk.

ICP-MS analysis of liver tissues collected four hours after administration of the last dose were analyzed using a two-way ANOVA, showing an interaction between gender and dose group for hepatic Cu concentration (F(2,4) = 24.93, *p* = 0.0055) and Ti concentration (F(2,4) = 8.446, *p* = 0.0367). While gender (Cu: F(1,2) = 3.57, *p* = 0.199; Ti: F(1,2) = 6.02, *p* = 0.134) and dose group (Cu: F(2,4) = 2.98, *p* = 0.161; Ti: F(2,4) = 1.35, *p* = 0.357) alone were not sources of variation. ICP-MS analysis demonstrated uptake of Cu in male pups (*p* = 0.0421), but not female pups (Figure 4). There was no increase in liver Ti concentration after TiO_2_ E171 administration.

### 3.6. Immune Cells in The Intestinal Tissue

**Figure 5:**
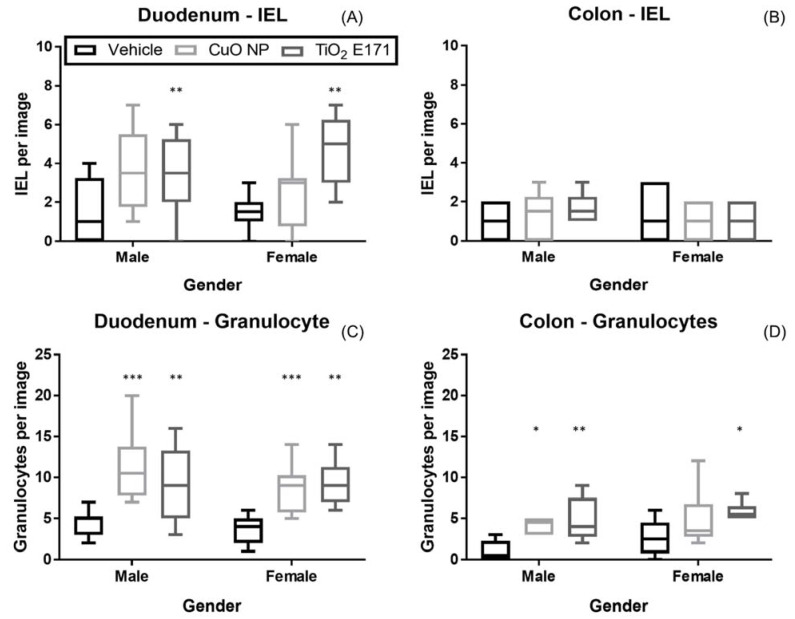
Immune cells, intraepithelial lymphocytes (IEL) (**A**,**B**) and granulocytes (**C**,**D**) located in H&E stained Swiss roll tissue sections of the duodenum (**A**, **C**) and colon (**B**, **D**) following oral administration of vehicle control, CuO NP, or TiO_2_ E171 in male and female pups at PND 10. Box and whisker plots of number of immune cells per image are shown. Statistical difference between vehicle control and dosing groups are noted by asterisk(s), *p*-value < 0.05 is noted with one asterisk, *p*-value < 0.01 with two asterisks, and *p*-value < 0.001 with three asterisks.

As the NPs transit through the lumen of the intestinal tract, they interact with the mucus layer and a small portion of the NPs reach the underlying epithelium. The question is what biological responses these interactions initiate. The changes in the number of intraepithelial lymphocytes (IEL) and granulocytes were evaluated in H&E stained Swiss roll tissue sections of the duodenum and colon (Figure 5). A two-way ANOVA was used to evaluate the role of gender and dose group on the number of immune cells in the intestinal tissues showing that dose group and not gender was the source of variance (Table 3). Following CuO NP administration, the number of granulocytes were significantly increased in both duodenum and colon for male and female pups (Figure 5C,D), while the number of IEL was not significantly increased by CuO NP administration (Figure 5A,B). The number of IEL was significantly increased following TiO_2_ E171 administration in duodenum in both male and female, but not in colon (Figure 5A,B). TiO_2_ E171 significantly increased the number of granulocytes in both duodenum and colon for male and female pups (Figure 5C,D). This increase in IEL and granulocytes demonstrates that NPs led to recruitment of immune cells, and the effect appears to be strongest in the small intestine and following administration of TiO_2_ E171.

## 4. Discussion

Little is known about the biotransformation, uptake, and biological responses to oral NP exposure in developing animals. Early life represents a vulnerable period and interference with the windows of critical development could have long-term health consequences. Several organs undergo considerable postnatal development, including the gastrointestinal tract and immune system. Thus, evaluating the biological effects of NP exposure in young animals postnatally is essential for understanding the potential health risks.

As the NPs pass through the gastrointestinal tract, they undergo biotransformation. Here the changes in PCPs, size, aggregation, and dissolution, were characterized for CuO NP and TiO_2_ E171 after in vitro simulated gastric digestion in rat pups at three different pre-weaning ages (PND 7, PND 14, and PND 21). Simulated human digestion, and the resulting impact on interactions between NPs and biological system, has been a focus of several in vitro studies. First presented as an in vitro digestion model for bioaccessibility of toxins [49], the simulated digestion method was later adopted in nanotoxicity studies [29,32,35,50]. While in vitro digestion provides valuable insight in the changes of NPs’ PCPs, it rests upon assumptions around oro-cecal transition time and fasting versus the presence of food matrix. It has been observed that a density dependent layering of food (e.g., liquid and solid food) takes place in the rat stomach [37,51,52]. This layering of the stomach content could be important for the gastric transit time of NPs, since aggregated NPs might settle on the bottom of the stomach and increase their gastric transit time. Here the simulated gastric digestion was evaluated for a range of time points; 1, 2, and 4 h.

Our findings demonstrated that increasing time of simulated gastric digestion increased the dissolution of CuO NP, while TiO_2_ E171, as expected, did not undergo any dissolution. However, both NPs displayed increased aggregation as a result of simulated digestion. These findings highlight the importance of considering different gastric transit times.

Overall, our results for gastric digestion of CuO NP in the acidic phase of postnatal rats agrees with the literature for copper (Cu) NP [36]. Cu NP (33 ± 11 nm) incubated for 24 h in artificial human gastric fluid (pH 1.5) led to 84.5% of the Cu NP to undergoing dissolution [36]. While we observed aggregation of digested food grade TiO_2_ E171 after simulated rat gastric digestion, another study of three different TiO_2_ NPs (22, 28, and 30 nm) digested in a standardized INFOGEST 2.0 in vitro digestion method, simulating human digestion, did not observe NP aggregation [30]. Bettencourt et al. [30] did not report dissolution data after simulated digestion of the three TiO_2_ NPs.

Since the oro-gastric transit time is influenced by a long list of factors including food matrix, exercise, fear and apprehension, acute and chronic diseases, trauma, drugs, and sleep, in addition to the possibility that more dense material (e.g., aggregated NPs) might settle at the bottom of the stomach and increase its gastric transit time, it is worth evaluating the changes in NP PCPs over a period of time. The findings presented here show that gastric fluids simulating the bland phase (~PND 7) and transitional phase (~PND 14) after four hours caused high levels of NP aggregation for both CuO NP and TiO_2_ E171, but limited dissolution for CuO NP (3.5–17%), while no dissolution for TiO_2_ E171 was detected. This suggests that the majority of CuO NP and TiO_2_ E171 orally administered to rat pups at age PND 7–10 should be particulates, especially since the pups were with the dam at all times and lactating.

Intestinal uptake of orally administered Al_2_O_3_ NP has been observed in rat pups dosed between PND 17–20 [14], but to the authors’ knowledge no reports of oral exposure or intestinal uptake of either CuO NP or TiO_2_ E171 exists for developing animals. Here intestinal uptake of CuO NP and TiO_2_ E171 was demonstrated using a combination of EDM/HSI evaluation of AB-PAS stained cross sections of the intestinal tract. In the small and large intestine tissue from both dosing groups, the highest percent of particles were found in the lumen, but low levels of particles were found in the epithelium demonstrating low levels of intestinal uptake of particles. The high level of particles in the lumen for both CuO NP and TiO_2_ E171 was supported by the simulated gastric digestion studies suggesting that a relatively small portion of CuO NP, and none of the TiO_2_ E171, underwent dissolution in the developing rat between age PND 7–10. ICP-MS analysis of liver tissues demonstrated intestinal uptake of Cu in male pups dosed with CuO NP. The lack of detectable levels of Cu in female pups dosed with CuO NP could either be due to gender-specific differences in uptake or tissue distribution. The level of Ti in liver tissue was not increased after administration of TiO_2_ E171. Gender-specific differences in intestinal uptake were also observed following orally administration of Al_2_O_3_ NP, showing a higher level of NP in the epithelium of the duodenum and ileum in female pups when dosed between PND 17–21 [14]. Together these two studies suggest that intestinal uptake of orally administered NPs is influence by both the NP PCPs and gender.

Oral uptake has been reported for adult animals administered CuO NP and Cu NP [36,53,54,55] and TiO_2_ NP [56,57,58,59]. Following orally administration of CuO NP (diameter 15–20 nm) to adult male rats for five consecutive days at concentrations of 32 and 64 mg/kg, significantly increased levels of Cu in the liver were detected by ICP-MS analysis, while Cu concentrations were only significantly increased in mesenteric lymph nodes for the high dose [54]. On the other hand, while oral administration of 100 mg/kg copper (Cu) NP (diameter 33 ± 11 nm) for 28 days did not lead to increased Cu concentration in blood or liver compared to control, a dose of 200 mg/kg did [55]. When Cu NP (diameter 33 ± 10 nm) was administered to adult male rats as a single oral dose of 500 mg/kg, the Cu concentration in blood was significantly increased (approximately 3-fold) after 24, 48, and 72 h post administration (but not at 12 h and 1, 2, and 4 weeks) [36]. Interestingly, the Cu concentrations in liver were significantly higher after 24, 48, 72 h, 1, and 2 weeks [36], suggesting that the clearance of Cu in the liver exceeds 2 weeks. Adult male rats receiving Cu NP (diameter 40–60 nm) formulated in feed for 4 weeks, with a Cu intake of 0.42 and 0.49 mg/5 days, also had a significantly increased concentration of Cu in the liver [53]. In adult animal intestinal uptake of oral administered TiO_2_ NP appears to be lower than for CuO NP, which is likely due to the neglectable dissolution of TiO_2_ NP in the intestinal fluids. Studies utilizing ICP-MS have not often detected elevated concentrations of Ti in analyzed tissues. Oral administration of TiO_2_ NP to adult rats for a duration of 30–90 days did not find any signs of oral uptake [60,61,62,63]. However, Talamini et al. [56] reported a significantly elevated concentration of Ti in liver after 3 days dosing per week over a period of 3 weeks of orally administered TiO_2_ E171 to male mice [56], while Li et al. [59] did not detect Ti in liver after 28 days of consecutive oral dosing of 100 mg/kg TiO_2_ NP in male mice, but found significantly increased concentrations in lung, kidney, and spleen [59]. These difference in oral uptake could possibly be a species-related difference between rats and mice. The presence of TiO_2_ E171 in intestinal tissue has been observed with imaging techniques in both male rats [57] and male mice [58], suggesting that intestinal uptake does happen in rats. Both studies observed TiO_2_ E171 in the small intestinal tract tissue including Peyer’s Patches using a combination of confocal microscopy and micro X-ray fluorescence (μ-XRF) [57,58], as well as TEM—energy dispersive X-ray analysis (EDX) [57,58], and nanoscale secondary ion mass spectrometry (nanoSIMS) imaging [57]. In the study presented here four, daily, consecutive oral doses of CuO NP between PND 7–10 mg/kg resulted in increased concentration of Cu in liver for male pups, but not for female pups. Furthermore, no increase in liver concentration of Ti was detected after TiO_2_ E171 administration. While microscopy analysis in this study showed low level of intestinal uptake for both genders dosed with CuO NP and TiO_2_ E171, only liver for CuO NP dosed males had an increased Cu concentration, suggesting gender specific and NP specific differences in intestinal uptake and tissue distribution. Overall, the data suggest a low level of intestinal uptake, with the majority of administered NPs passing through the intestinal tract demonstrating that the mucus layer presents an efficient barrier.

Despite the mucus layer being an efficient barrier and low levels of intestinal uptake of CuO NP and TiO_2_ E171 were observed, the number of immune cells, IEL and granulocytes, in the small and large intestinal tract were found to be significantly increased. IEL comprise both natural and induced IELs and are the first line of defense in the intestinal tract [64]. Granulocytes are part of the innate immune response, and include neutrophils, eosinophils, and basophils, their main function being to eliminate microbes that cross the epithelial barrier. It is therefore concerning that orally administered TiO_2_ E171 leads to a significant increase in IEL and orally administrated CuO NP and TiO_2_ E171 lead to significant increase in granulocytes in the intestinal tract of developing rats, since it suggests an imbalance in the intestinal homeostasis. The intestinal homeostasis depends on three major components, the epithelium, the microbiome, and the immune cells. Increased numbers of scattered individual granulocytes and IEL are suggestive of intestinal irritation, immune stimulation, or minimal inflammation, since histopathological evaluation of the H&E stained tissue sections did not show any sign of active inflammation. TiO_2_ NP has also been found to increase the number of non-allergenic mast cells in the stomach tissue in young rats (3 weeks of age at the beginning of study) following 30 days of oral administration of 200 mg/kg [60]. IEL and granulocytes are important for maintaining intestinal homeostasis, and their role in intestinal dysfunction including inflammatory bowel disease (IBS), Crohn’s disease, and celiac disease is a topic of investigation [64,65,66,67,68]. Together these results suggest that both CuO NP and TiO_2_ E171 interfere with the immune cells in the gastrointestinal tract following early life exposure and emphasize the need to investigate the long-term effect on intestinal homeostasis.

## 5. Conclusions

A carefully timed and coordinated series of sequential developmental events starting prenatal and continuing postnatal is required for the successful establishment of the mammalian immune system [27]. Interference with these series of events can lead to long-term immune dysfunctions. We show here that four orally administered doses of 10 mg/kg CuO NP and 10 mg/kg TiO_2_ E171 administered between PND 7–10 led to low levels of intestinal NP uptake and an increase in immune cells in the intestinal tract. Furthermore, as the health and architecture of the intestinal epithelium has been reported to be adversely affected in adult male rats by oral exposure to CuO NP [53] and TiO_2_ NP [57,69] it is necessary to understand the long-term effect of early life exposure on the intestinal homeostasis.

## Figures and Tables

**Table 1:** Chemical formulation of in vitro rat gastric fluid reflecting different postnatal ages tested in this study and time (h) of digestions.

Gastric Phase	Bland	Transitional	Acidic
Age	~PND 7	~PND 14	~PND 21
pH	7.0	6.0	4.0
Chemical Formulation	0.315 g/L NaHCO_3_	0.13 g/L Pepsin (from porcine gastric mucosa)	0.27 g/L Pepsin (from porcine gastric mucosa)
	8.78 g/L NaCl	1.5 g/l Mucin	1.5 g/l Mucin
		Hydrochloric acid	Hydrochloric acid
		0.315 g/L NaHCO_3_	0.315 g/L NaHCO_3_
		8.78 g/L NaCl	8.78 g/L NaCl
Incubation Time	1, 2, and 4 h	1, 2, and 4 h	1, 2, and 4 h

**Table 2:** In vitro gastric digestion of CuO NP and TiO_2_ E171 E171 at different postnatal ages and gastric transit times.

			DLS ^a^		ICP-OES ^b^	
Gastric Phase (Age)	Solution	Inc. Time (h)	CuO NP Diameter [nm], Average ± std. (PdI)	TiO_2_ E171 Diameter [nm], Average ± std. (PdI)	CuO NP %Cu Digested in Filtrate	TiO_2_ E171 %Ti Digested in Filtrate
Control	DI water	NA c	441 ± 68.2 (0.37)	322 ± 38.3 (0.24)	ND d	ND
Bland Phase (~PND 7)	Gastric fluid, pH 7	1	348 ± 52.1 (0.47)	1207 ± 139 (0.82)	ND	ND
		2	1151 ± 149 (0.64)	1292 ± 144 (0.96)	ND	ND
		4	2044 ± 484 (0.92)	1987 ± 341 (1.0)	3.49 ± 1.77	ND
Transitional Phase (~PND 14)	Gastric fluid, pH 6	1	1237 ± 103 (0.66)	1122 ± 81.4 (0.68)	ND	ND
		2	1151 ± 96.5 (0.74)	1280 ± 127 (0.79)	4.86 ± 1.81	ND
		4	1813 ± 41.0 (0.96)	1377 ± 179 (0.88)	17.4 ± 4.53	ND
Acidic Phase (~ PND 21)	Gastric fluid, pH 4	1	841 ± 15.2 (0.62)	779 ± 56.8 (0.68)	24.7 ± 3.92	ND
		2	679 ± 62.6 (0.81)	821 ± 103 (0.70)	52.6 ± 4.62	ND
		4	1365 ± 39.0 (0.75)	1448 ± 224 (0.69)	78.2 ± 5.85	ND

^a^ DLS measurements of the hydrodynamic diameter (nm) (mean ± standard deviation [std.]) and polydispersity index (PdI) results for CuO NP and TiO_2_ E171 incubated in in vitro gastrointestinal fluids. ^b^ ICP-OES results for CuO NP and TiO_2_ E171 dissolution following incubated in in vitro gastrointestinal fluids, expressed as percent digested metal ions in filtrate compared to initial metal ion concentration. c NA = not applicable. d ND = not detected.

**Table 3:** Two-way ANOVA was used to analyse the effects of gender and dose group on the number of intestinal immune cells.

	Duodenum ^b^	Colon ^b^
IEL ^a^	Gender: F(1,9) = 0, *p* > 0.9999	Gender: F(1,5) = 0.4, *p* = 0.5549
	Dose group: F(2,18) = 10.62, *p* = 0.0009 *** ^c^	Dose group: F(2,10) = 0.05714, *p* = 0.9448
	Gender x Dose group: F(2,18) = 2.648, *p* = 0.0981	Gender x Dose group: F(2,10) = 0.8235, *p* = 0.4666
	Gender: F(1,9) = 1727, *p* = 0.2213	Gender: F(1,5) = 1.969, *p* = 0.2196
Granulocytes	Dose group: F(2,18) = 17.92, *p* < 0.0001 *** ^c^	Dose group: F(2,10) = 5.317, *p* = 0.0267 * ^c^
	Gender x Dose group: F(2,18) = 1.863, *p* = 0.1840	Gender x Dose group: F(2,10) = 0.5512, *p* = 0.5928

^a^ Intraepithelial lymphocytes (IEL); ^b^ H&E stained Swiss roll tissue sections of the duodenum and colon at PND 10; ^c^ Statistical difference between vehicle control and dosing groups are noted by asterisk(s), *p*-value < 0.05 is noted with one asterisk, and *p*-value < 0.001 with three asterisks.

## Data Availability

Data is contained within the article or Supplementary Material.

## Supplementary Materials

The following are available online at https://www.mdpi.com/article/10.3390/nano11061487/s1. Figure S1: Spectral libraries for each NP and number of spectra per library, Figure S2: TEM images of CuO NP and TiO_2_ E171, Figure S3: SEM images of in vitro gastric digested CuO NP and TiO_2_ E171. Table S1: ICP-OES analysis of CuO NP and TiO2 E171 in the solutions involved in tissue fixation, preparation and processing. Table S2: In vivo dose formulation of CuO NP and TiO_2_ E171 characterization.
