# Supplementary material for: Simulated Gastric Digestion and In Vivo Intestinal Uptake of Orally Administered CuO Nanoparticles and TiO2 E171 in Male and Female Rat Pups
Source: Nanomaterials (Basel). 2021 Jun 4;11(6):1487. doi: 10.3390/nano11061487 (PMC8230348; doi:10.3390/nano11061487)
Supplement: Supplementary file 1 [file nanomaterials-11-01487-s001.zip › nanomaterials-1232770-supplementary.pdf]

Simulated Gastric Digestion and *In Vivo* Intestinal Uptake of Orally Administered CuO Nanoparticles and TiO_2 E171 in Male and Female Rat Pups

Ninell P. Mortensen[1,*], Maria Moreno Caffaro[1], Shyam Aravamudhan[2], Lakshmi Beeravalli[2], Sharmista Prattipati[2], Rodney W. Snyder[1], Scott L. Watson[1], Purvi R. Patel[1], Frank X. Weber[1], Stephanie A. Montgomery[3], Susan J. Sumner[4], Timothy R. Fennell[1]

Figure S1. Spectral libraries for each NP and tissue. The number of spectra per library is listed.

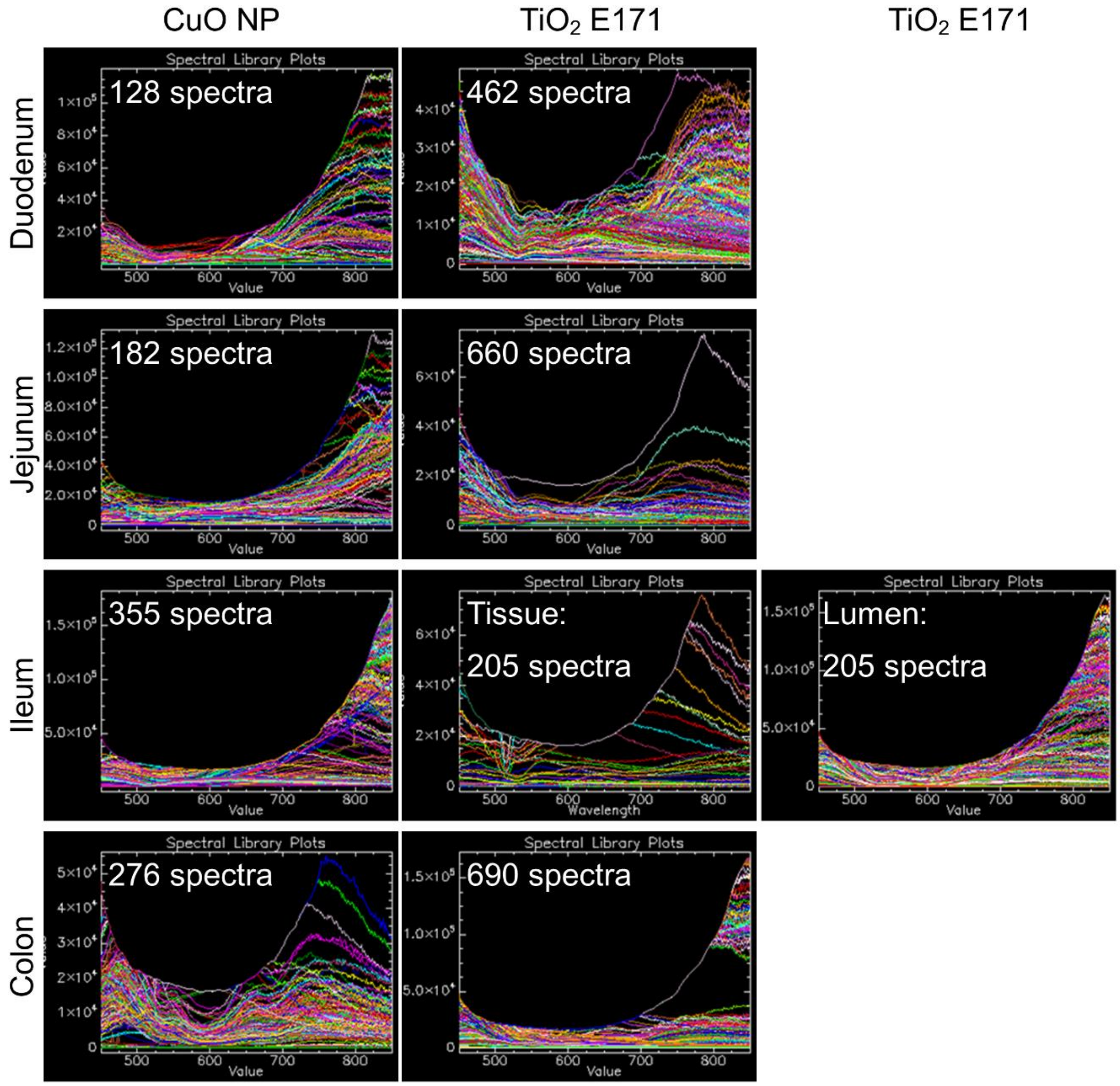

Figure S2. TEM images of (A) CuO NP and (B) TiO_2 E171 with scale bar.

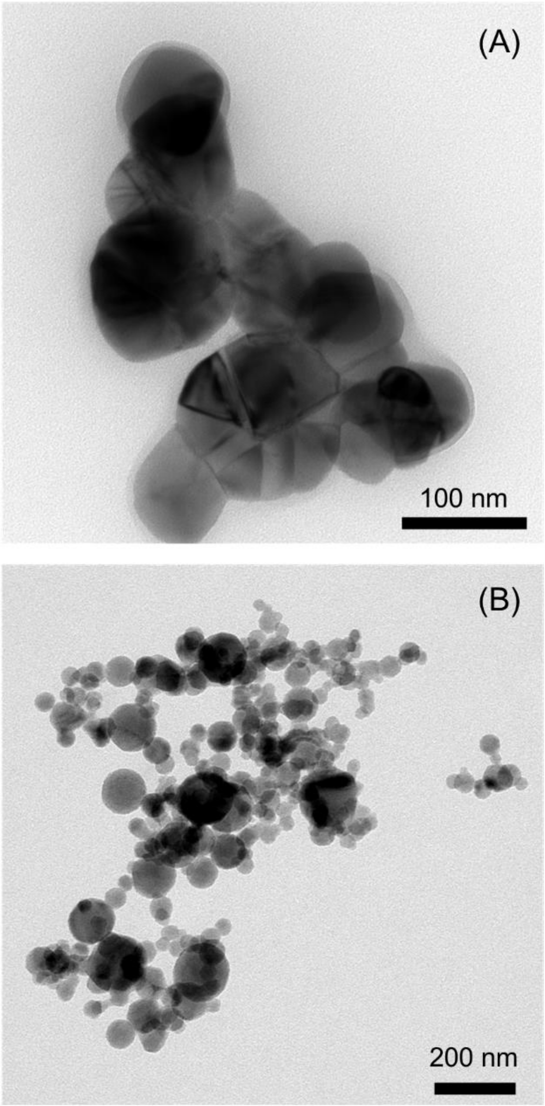

Figure S3. Simulated *in vitro* rat gastric digestion (4 h) of CuO NP and TiO_2 E171 in gastric fluids reflecting different ages: bland phase ~PND 7 (pH = 7), transitional phase ~PND 14 (pH = 6), and acidic phase ~PND 21 (pH = 4).

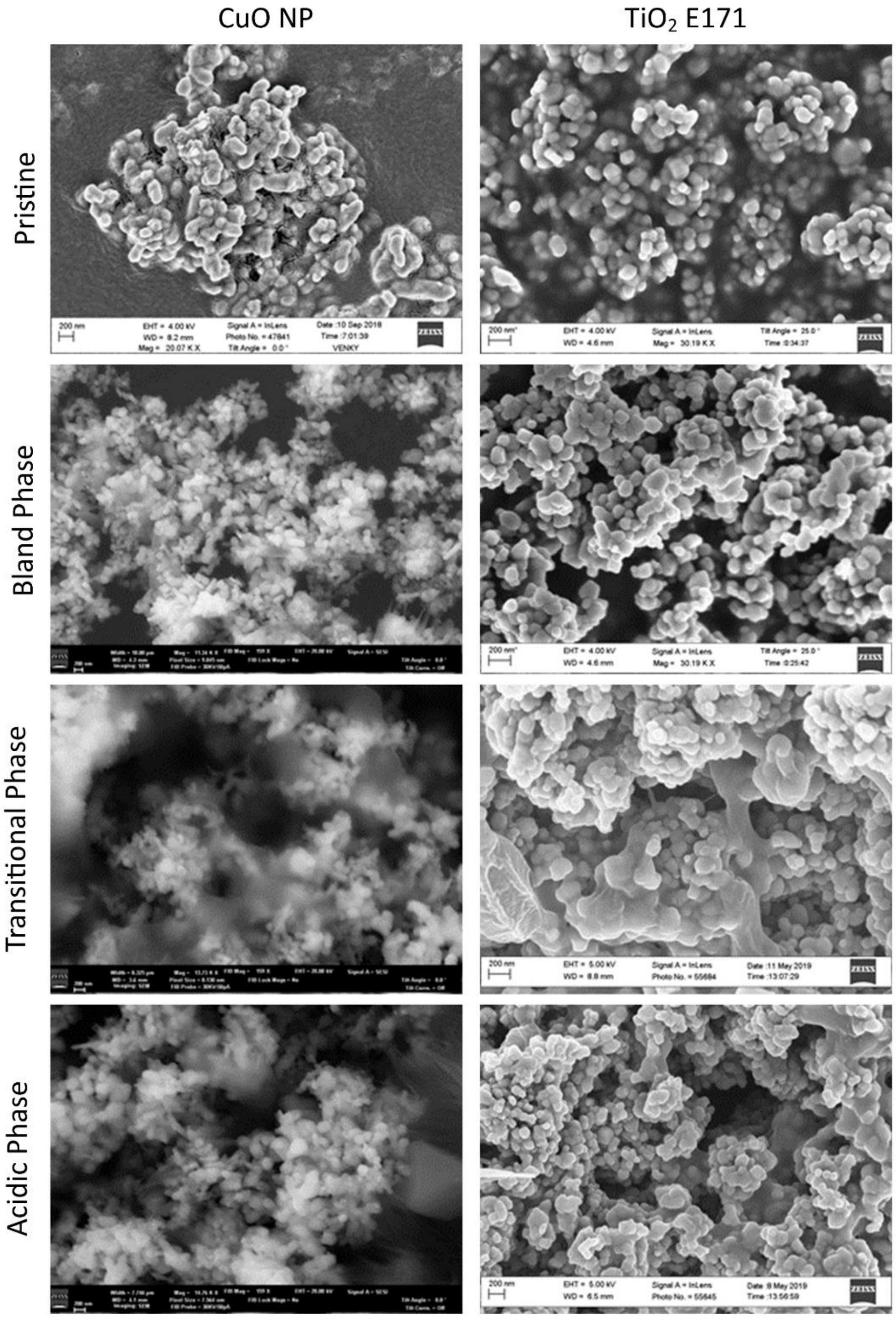

Table S1. ICP-OES analysis of CuO NP and TiO_2 E171 in the solutions involved in tissue fixation, preparation and processing. ND = not detected.

Solution	Time Point	%Cu digested in filtrate (compared to initial Cu)	%Ti digested in filtrate (compared to initial Ti)
10% formalin	24 h	0.85 ± 0.25	ND[a]
	72 h	0.97 ± 0.34	ND
Bouin's fixative	10 min	ND	ND
	30 min	ND	ND
Metha-Carnoy's solution	2 h	ND	ND
	4 h	1.39 ± 0.47	ND
Ethanol – 70%	10 min	ND	ND
	30 min	ND	ND
Ethanol – 90%	10 min	ND	ND
	30 min	ND	ND
Ethanol – 100%	10 min	ND	ND
	30 min	ND	ND
Xylene	10 min	ND	ND
	30 min	ND	ND

[a]ND = not detected.

Table S2. *In vivo* dose formulation of CuO NP and TiO_2 E171 characterization.

		DLS[a]		Zeta potential	NTA[b]	
ENM	Time point (h)	Hydrodynamic diameter (nm)	Polydispersity index (PdI)	mV	Mean diameter (nm)	Mode (nm)
CuO NP	0	1,582 ± 99.0	0.551	-10.7 ± 0.726	237 ± 99.0	183
	4	1,498 ± 102	0.521	-19.5 ± 0.497	261 ± 73.0	196
TiO_2 E171	0	289 ± 3.87	0.261	-0.142 ± 1.27	85.0 ± 40.0	71
	4	300 ± 9.91	0.334	0.992 ± 0.518	250 ± 109	107

[a]DLS measurements of the hydrodynamic diameter (nm) (mean ± standard deviation [std.]) and polydispersity index (PdI).

[b]NTA measurements of the mean diameter (nm) (mean ± standard deviation [std.]) and mode (nm).
